# The effect of performance-related pay of hospital doctors on hospital behaviour: a case study from Shandong, China

**DOI:** 10.1186/1478-4491-3-11

**Published:** 2005-10-27

**Authors:** Xingzhu Liu, Anne Mills

**Affiliations:** 1 Abt Associates Inc., Bethesda, Maryland, USA; 2 Health Economics and Financing Programme, London School of Hygiene and Tropical Medicine, London, UK

## Abstract

**Background:**

With the recognition that public hospitals are often productively inefficient, reforms have taken place worldwide to increase their administrative autonomy and financial responsibility. Reforms in China have been some of the most radical: the government budget for public hospitals was fixed, and hospitals had to rely on charges to fill their financing gap. Accompanying these changes was the widespread introduction of performance-related pay for hospital doctors – termed the "bonus" system. While the policy objective was to improve productivity and cost recovery, it is likely that the incentive to increase the quantity of care provided would operate regardless of whether the care was medically necessary.

**Methods:**

The primary concerns of this study were to assess the effects of the bonus system on hospital revenue, cost recovery and productivity, and to explore whether various forms of bonus pay were associated with the provision of unnecessary care. The study drew on longitudinal data on revenue and productivity from six panel hospitals, and a detailed record review of 2303 tracer disease patients (1161 appendicitis patients and 1142 pneumonia patients) was used to identify unnecessary care.

**Results:**

The study found that bonus system change over time contributed significantly to the increase in hospital service revenue and hospital cost recovery. There was an increase in unnecessary care and in the probability of admission when the bonus system switched from one with a weaker incentive to increase services to one with a stronger incentive, suggesting that improvement in the financial health of public hospitals was achieved at least in part through the provision of more unnecessary care and drugs and through admitting more patients.

**Conclusion:**

There was little evidence that the performance-related pay system as designed by the sample of Chinese public hospitals was socially desirable. Hospitals should be monitored more closely by the government, and regulations applied to limit opportunistic behaviour. Otherwise, the containment of government financing for public facilities may result in an increase in the provision of unnecessary care, an increase in health costs to society, and a waste in social resources.

## Background

Health policy researchers and policy makers have increasingly recognized that health care providers have a powerful influence on health care provision and the use of health care resources. With the recognition that public hospitals are often productively inefficient, reforms have taken place worldwide to increase the administrative autonomy and financial responsibility of public hospitals [1,2]. Reforms in China are perhaps some of the most radical. Starting from the early 1980s, the government budget for public hospitals was fixed, and hospitals had to rely on charges to fill the gap between hospital expenditure and income from the government. Medical prices regulated by government were increased and hospitals allowed to earn profits from certain services and from drugs. Accompanying these changes was the widespread introduction of performance-related pay for hospital doctors – termed the "bonus" system. The policy objective was to improve productivity and cost recovery [3].

The bonus system is widespread and used by almost all hospitals in China. The types of hospital bonus system can be summarized into three different forms: flat bonus, quantity-related bonus and revenue-related bonus [4-12]. A flat bonus is distributed among hospital staff equally or almost equally, with the amount depending on the overall financial status of the hospital. A quantity-related bonus is paid according to the quantity of services provided (visits, admissions, inpatient days, medical procedures, and tests and examinations), usually with a quantity target above which the bonus is paid. A revenue-related bonus depends on the revenue generated by doctors through provision of services and drugs over a revenue target.

A survey of bonus systems in all county hospitals in Shandong province in 1997 [13] found that 78% had a revenue-related system and the remaining 22% a quantity-related system. The average bonus per month (83 yuan) was around 10% of the monthly salary but depended on the financial status of the hospital. In 1997, 11% of hospitals paid no bonus, and 37% paid an average bonus of over 100 yuan per month. Average bonus amounts were not significantly different by type of bonus system.

The objectives of the study reported here were to assess the effects of the bonus system on hospital revenue, cost recovery and productivity, and to explore whether bonus pay was associated with the provision of unnecessary care. The primary hypothesis tested in the study was that the impact of a bonus system on revenue, cost recovery, productivity and unnecessary care would depend on the strength and direction of the economic incentive of different bonus systems, and doctors' responses to these incentives. It was assumed that an increase in provision of necessary care would provide utility gains for doctors in the form of income and a utility loss in the form of greater effort, and that the provision of unnecessary care would also provide income utility gains, but at a utility loss stemming from ethical concerns as well as greater effort. Thus the behaviour of doctors would be determined by the trade-off of utility gains and losses, and the motivation for doctors to provide unnecessary care would be constrained by their desire for leisure and for ethical behaviour.

It was hypothesized that a flat bonus might motivate doctors to provide necessary care but would be less likely to motivate them to provide unnecessary care. If there is enough demand, the effort of an individual doctor to provide more necessary care would benefit patients, the hospital and the doctor as well. Productivity, cost recovery and quality of care could all improve. If there is insufficient demand, the doctor could induce demand but might be less motivated to do so because the bonus income would be distributed throughout the hospital. A quantity-related bonus would provide a stronger economic incentive for doctors to provide a greater quantity of services, and might help to improve productivity, cost recovery and quality of care if there is sufficient demand. But when demand is insufficient, doctors may be motivated to induce demand by providing more care, regardless of need. What types of service would be overprovided would depend on how quantity is defined and measured. Finally, a revenue-related bonus provides the strongest incentive for doctors to induce patient demand for both services and drugs.

## Methods

### Definition and measurement of key indicators

Productivity is generally defined as the ratio of a hospital's output to its input. This study employed both unidimensional ratio analysis (outpatient visits per doctor, admissions per doctor, bed occupancy rate, length of stay); and Data Envelopment Analysis, a linear programming method that measures the technical efficiency of production [14] and has been used to measure hospital productivity by numerous authors (e.g. [15]). The relative level of hospital productivity was indicated by the DEA efficiency score, which ranges from 0 to 100, with, for a given set of inputs, 0 meaning no output and 100 meaning the maximization of output. The objective function of hospital *o *compared with the *n *hospitals in the data set is:



where *o *represents the hospital being evaluated in the set of j = 1,...,n hospitals; *E *is the efficiency score; *u*_*r *_is the weight for the *rth *output; *y*_*ro *_is the *rth *output for the *oth *hospital; *s *is the number of outputs; *v*_*i *_is the weight of the *ith *input; *x*_*io *_is the *ith *input for the *oth *hospital; *m *is the number of inputs. The DEA efficiency score was calculated using software developed by Warwick Business School [16], assuming constant returns to scale. Hospital inputs included the number of doctors, the number of nurses, the monetary value of hospital fixed assets, the number of hospital beds and the monetary value of supplies. The hospital outputs included the number of admissions, outpatient visits and surgical operations.

Cost recovery was defined as service revenue expressed as a percentage of recurrent and total costs. Unnecessary care was defined as services and drugs provided that were judged by a panel of doctors to lead to no improvement in patient outcomes. Unnecessary care can be assessed only in relation to the nature of the cases being treated, so appendicitis and child pneumonia were selected as tracer diseases on the basis that they were common so there were enough cases for each hospital and year; they had clear-cut diagnoses so the sample would be homogeneous; and both had a standard plan of treatment, so variation in treatment would not be due to treatment uncertainty. Six surgeons (for appendicitis cases) and six paediatricians (for pneumonia cases) worked together with the investigators to develop guidelines for appropriate management of the two tracer diseases and for identification of unnecessary care. These guidelines were positive lists of types and quantities of services and drugs necessary for the improvement of the health outcomes of patients. The unnecessary care indicator was unnecessary care expenditure as a percent of total expenditure. A detailed description of the methodology is in Liu and Mills [17].

### Samples and data

Based on a census of all 127 county general hospitals in Shandong province [18], 25 hospitals were selected that had experienced change of bonus system and that had complete inpatient files for the previous 10 years. These hospitals were categorized into three groups based on county income level, and two hospitals randomly selected from each group. Data were collected from these six panel hospitals for the period 1978–1997 on the type of bonus system in different years; total revenue by source; recurrent and capital cost; staff numbers; and activity data (outpatients, inpatients, operations, CT scans, average length of stay, bed occupancy rate).

Inpatient records were collected from the six panel hospitals for each change of bonus system and encompassing the year of the switch and the two years before and after. Inpatient records for each year, disease and hospital were drawn from the beginning of the year until the sample size reached 30 or until there were no more records available. Altogether the study included 1161 appendicitis patients and 1142 pneumonia patients.

Patient files were randomly distributed to the relevant doctors who reviewed the files and recorded the types and quantities of services and drugs actually used, and the types and quantities of unnecessary services and drugs. If the removal of unnecessary items was considered to result in inadequate treatment, substitute necessary services and drugs were added in. Finally, actual expenditures and unnecessary expenditures were computed by accountants according to the 1997 provincial fee schedule.

Because of the burden of work, each record was reviewed by only one doctor, but to check for consistency, 61 patient records for appendicitis and 57 for pneumonia were selected and randomly distributed to the panel doctors for re-reviews, without their knowledge, and the results compared. Means were very similar: none of the *p*s of t-tests were less than 0.05 and most were very close to 1.

### Data analysis

In the data analysis, we first described the historical changes in bonus system, hospital revenue, productivity and cost recovery, and then analysed the level of unnecessary care.

Trend analysis was performed to examine the changes in hospital revenue, cost recovery, productivity, and the rate of unnecessary expenditure following the bonus switches. In the trend analysis, the indicators were assessed for a continuous five years for each of the hospital bonus switches, including the year of bonus switch and the two years before and the two years after the switch.

In correlation and regression analysis, we examined the relationships of the bonus system with the four key variables (hospital revenue, cost recovery, hospital productivity and unnecessary care). First, the interrelationships among these four variables were examined through correlation analysis of each pair of variables. The observation units were hospital-years. The type of bonus was measured by a dummy variable with values reflecting the expected strength of economic incentives to overprovide (non-bonus = 1; flat bonus = 2; revenue-related bonus = 3). Cost recovery was measured by the rate of recovery of recurrent cost, and hospital productivity by the DEA efficiency score.

The subsequent set of analyses used stepwise regression analysis to examine whether and how hospital revenue, cost recovery, unnecessary care and productivity were related to each other, and to what extent they were explained by the bonus system. Each of the four variables was taken in turn as a dependent variable, and the factors that might explain the variations in the dependent variable examined (the results of the four stepwise regression analyses yielded results similar to a general regression analysis in terms of R-squares and statistical significance levels of the independent variables). Besides indicators of revenue, cost recovery, unnecessary care and productivity, the year, names of hospitals and bonus type were put into the regression models as independent variables. The six hospital names were arranged into five dummy variables. The year measured all the factors that changed with time (e.g. medical price inflation, technology improvement and the increase in demand for care). The names of hospitals measured all the factors that were related to each hospital (e.g. the level of demand faced by an individual hospital, management capacity, degree of observance of ethical codes, etc.).

## Results

### Historical changes in bonus system, hospital revenue, productivity and cost recovery

All six panel hospitals had experienced bonus switches from no-bonus to flat-bonus and then to revenue-related bonus (Table 1). None had experience of quantity-related bonus. Before the early 1980s, no hospital had a bonus system; by 1988 all hospitals had a flat bonus system; and by the middle of the 1990s, all hospitals had a revenue-related bonus system.

**Table 1:** Bonus systems of the six panel hospitals (1978 – 1997)

**Year**	**Zhaoyuan**	**Liangshan**	**Qixia**	**Weishan**	**Changyi**	**Yanzhou**
1978	1	1	1	1	1	1
1979	1	1	1	1*	1	1
1980	1	1	1*	1*	1	1
1981	1	1	1*	2*	1	1
1982	1	1	2*	2*	*1*	1*
1983	1	1	2*	2*	*1*	1*
1984	1	1	2*	2	*2*	2*
1985	*1*	1	2	3	*2*	2*
1986	*1*	*1*	2	3	*2*	2*
1987	*2*	*1*	2	3	2	2
1988	*2*	*2*	2	3	2	2
1989	*2*	*2*	2	3	2	2
1990	2	*2*	2	3	2	2
1991	*2*	*2*	*2*	*3*	2	2
1992	*2*	*2*	*2*	*3*	*2*	*2*
1993	*3*	*2*	*3*	*1*	*2*	*2*
1994	*3*	*3*	*3*	*1*	*3*	*3*
1995	*3*	*3*	*3*	*3*	*3*	*3*
1996	3	*3*	3	*3*	*3*	*3*
1997	3	3	3	*3*	3	3

Notes:

1) 1: No bonus; 2: Flat bonus; 3: Revenue-related.

2) Numbers in italics indicate years for which inpatient records were collected.

3) * indicates data on tracer diseases were not available around the years of the bonus switch.

Coinciding with the change in bonus systems, over the period 1978–97, there was a remarkable increase in hospital revenue, an increase in hospital cost recovery, a doubling of admissions, a decrease in visits and a tripling of operations (Table 2). The average revenue increase in real terms was 16.3% per year, and 3.7% per year for admissions. In 1978, only 3% of outpatients were admitted into hospital and only 17% of inpatients were operated on, while by 1997 these had increased to 7% and 25%. Staff numbers and hospital beds increased over time and because the increase in inputs exceeded the increase in outputs, most productivity indicators decreased (Table 3) with the exception of operations per doctor. Quality changes might have occurred but could not be assessed with the available data.

**Table 2:** Changes in average activity levels of the panel hospitals, 1978 – 1997

**Year**	**Change in revenue (1975 = 100)**	**Recovery of recurrent cost (%)**	**Change in the number of: (1975 = 100)**			
				
			**Admissions**	**Visits**	**Operations**	**No. of visits per admission**
1978	100	75.4	100	100	100	31.7
1979	124	79.3	111	102	103	29.3
1980	132	79.3	123	105	116	27.1
1981	140	82.0	124	106	114	27.2
1982	155	82.1	104	108	118	32.9
1983	168	80.3	110	105	122	30.3
1984	206	77.9	106	112	131	33.4
1985	206	73.3	108	111	120	32.4
1986	270	82.7	119	115	141	30.6
1987	341	89.1	138	121	161	27.9
1988	436	97.8	149	120	181	25.6
1989	525	104.9	161	112	196	22.0
1990	634	101.4	160	113	202	22.5
1991	728	93.8	171	120	220	22.3
1992	839	98.3	149	115	218	24.6
1993	1,046	100.2	157	97	245	19.6
1994	1,063	90.8	158	85	254	17.0
1995	1,280	99.4	172	83	265	15.2
1996	1,552	104.1	185	90	268	15.4
1997	1,755	102.0	201	91	283	14.3

**Table 3:** Changes in productivity in the panel hospitals, 1978 – 1997

**Year**	**Bed occupancy rate (%)**	**Length of stay (days)**	**Visits per doctor**	**Admissions per doctor**	**Operations per doctor**	**DEA efficiency score**
1978	86	9.1	2041	64.3	11.3	97.1
1979	91	9.0	1955	68.7	10.9	97.1
1980	88	9.0	1893	72.0	11.5	96.9
1981	90	9.2	1712	64.0	10.2	95.6
1982	89	10.0	1660	51.2	9.8	87.9
1983	90	10.2	1561	52.0	9.8	86.5
1984	87	10.5	1480	43.5	9.6	84.8
1985	91	10.7	1374	41.9	8.2	74.7
1986	94	11.3	1378	44.9	9.3	79.5
1987	93	11.2	1289	44.6	9.0	77.0
1988	95	11.1	1297	48.4	10.5	75.2
1989	92	11.3	1063	45.6	9.9	77.1
1990	94	11.8	1066	45.1	10.3	74.9
1991	92	11.1	1165	50.3	11.6	78.9
1992	88	12.2	1019	39.6	10.4	76.2
1993	86	11.7	839	40.9	11.4	75.1
1994	79	11.3	720	40.2	11.3	74.8
1995	82	10.8	675	42.2	11.4	74.7
1996	82	10.2	689	44.2	11.1	73.5
1997	79	10.6	684	48.2	11.8	72.6

### Unnecessary expenditure

There was a large amount of unnecessary expenditure for both tracer diseases. The average expenditure (1997 prices) for an appendicitis patient was 774 yuan (USD 95), of which 18% was considered unnecessary. For a pneumonia patient these figures were 559 yuan (USD 68) and 19%. Further analyses showed that more than one third of the expenditure for drugs was deemed unnecessary for both appendicitis (38%) and pneumonia (34%), and this made up 49% (appendicitis) and 73% (pneumonia) of total unnecessary expenditure. Unnecessary expenditure for doctors' and nurses' services, associated with excessive lengths of stay, made up the second largest share of unnecessary expenditure, accounting for 43% (appendicitis) and 21% (pneumonia). In terms of expenditure for doctors' and nurses' services, 13% (appendicitis) and 9% (pneumonia) of this was unnecessary. Although more than 50% of the expenditure for examinations for appendicitis patients was unnecessary, it made up only about 1% of the total unnecessary expenditure since little was spent on examinations. Unnecessary expenditure for laboratory tests was small in terms of both its contribution to total unnecessary expenditure (1%), and its percentage of expenditure for tests (7%).

### The relationship of bonus switch to hospital revenue, cost recovery, productivity and unnecessary care: trend analysis

The above data show that there had been considerable changes over time in hospital revenue and productivity, and also that there was a considerable amount of unnecessary care. But were these features related? This section examines this question through analysis of trends; the subsequent section examines the question through correlation and regression analysis.

Table 4 summarizes the changes in key indicators by hospital and type of bonus switch. When three hospitals changed their bonus system from non-bonus to flat bonus:

**Table 4:** Changes in revenue, cost recovery, productivity and unnecessary care with changes in bonus system

**Name of hospital**	**Rate of change of revenue**	**Rate of cost recovery**	**Visits per admission**	**Admissions per operation**	**DEA efficiency score**	**Rate of unnecessary care**
***Switch from non bonus to flat bonus***						
Zhaoyuan	Increase	Increase	Decrease	Increase	Decrease	Increase
Liangshan	Increase*	Increase*	Decrease	Increase	Decrease	Decrease
*Changyi*	Increase	Increase*	Increase	Decrease	Decrease	Increase
***Switch from flat to revenue-related bonus***						
Zhaoyuan	Increase	Increase	Decrease	Decrease	Increase	Increase
Liangshan	Increase*	Increase*	Decrease	Decrease	Decrease	Decrease
Qixia	Increase	Decrease	Decrease	Decrease	Increase	No change
Changyi	Increase	Decrease	Decrease	Decrease	Increase	Increase
Yanzhou	Increase	Increase	Decrease	Decrease	Increase	Decrease
Weishan	Increase	Increase	Decrease	Decrease	Increase	Increase
***Switch from revenue-related to flat bonus***						
Weishan	Decrease	Increase	Decrease	Increase	Decrease	Decrease

• the rate of growth of revenue increased in all three;

• cost recovery increased in all three;

• two hospitals out of three showed a decrease in the visits/admission ratio, meaning that more patients were admitted out of those attending for outpatient care;

• one hospital out of three showed a decrease in the admissions/operation ratio, meaning that a higher share of inpatients were operated on;

• productivity decreased in all three;

• unnecessary care increased in two out of three.

When hospitals conducted a further switch from flat bonus to revenue-related bonus:

• all six hospitals increased their rate of growth of revenue

• the majority (4/6) showed a decrease in cost recovery

• all six hospitals showed a decrease in the visits/admission ratio

• all six hospitals showed a decrease in the admissions/operation ratio

• five of the six hospitals experienced increases in productivity

• half showed an increase in unnecessary care.

Despite some inconsistent results, likely to reflect location-specific factors, an overall pattern emerges. First, the implementation of a flat bonus where previously there had been no bonus system seemed to be associated with an increase in hospital revenue and cost recovery, a decrease in hospital productivity and an increase in unnecessary care. Second, the implementation of a revenue-related bonus following a flat bonus system appeared to have increased hospital revenue and cost recovery, encouraged higher admissions and operations rates, and increased hospital productivity, with an unclear effect on unnecessary care.

### The interrelationships among bonus system, hospital revenue, cost recovery, productivity and unnecessary care

Results of correlation analysis (the correlation coefficients and their *p *values for the null hypothesis that the correlation coefficients are zero) are shown in Table 5. Bonus type was negatively correlated with DEA efficiency score (*p *< 0.01) but positively correlated with unnecessary care (*p *< 0.05), with service revenue (*p *< 0.01) and the rate of cost recovery (*p *< 0.01). These results mean that with a change in the bonus payment from non-bonus to flat bonus and to revenue-related bonus, hospital revenue increased and cost recovery improved. However, the improvement in financial status was associated with reductions in hospital productivity and increase in unnecessary care.

**Table 5:** Correlations among bonus system, productivity, cost recovery and unnecessary care

	**Type of bonus**	**Efficiency score**	**Service revenue**	**Cost recovery**
Efficiency score	-0.4075			
	(0.001)			
Service revenue	0.7353	-0.2952		
	(0.001)	(0.001)		
Cost recovery	0.3002	-0.2559	0.3328	
	(0.009)	(0.005)	(0.0002)	
Unnecessary care	0.3502	-0.2989	0.2374	0.0534
	(0.012)	(0.035)	(0.0969)	(0.7128)

Sample sizes: n = 50 for the correlation with unnecessary care; for all the others n = 120

The negative correlations between hospital productivity and cost recovery (*p *< 0.01) and hospital productivity and hospital revenue (*p *< 0.01) are consistent with the previous trend analysis, which showed an increase in cost recovery (and revenue) but a decrease in hospital productivity over time. Reduction in hospital productivity may have resulted from increased competition between providers, which encouraged county hospitals to provide more drugs and services per patient and to charge them more in order to increase revenue, and/or the increasing cost may have deterred people from seeking care.

Hospital productivity and unnecessary care were negatively correlated (*p *< 0.05). This relationship implies that if hospitals provide less unnecessary care and the expenditure of patients is therefore less, the hospital will be able to attract more patients. This is theoretically as expected because reduction in price will lead to increase in patient demand.

The relationships were not statistically significant between unnecessary care and hospital revenue, and unnecessary care and cost recovery (*p *> 0.05 for both). Individual hospital analysis showed that when the bonus switched from non-bonus to flat bonus or from flat bonus to revenue-related bonus, both unnecessary care and cost recovery tended to increase. In theory, there should indeed be a positive relationship between unnecessary care and hospital cost recovery. The reason why the relationship was not statistically significant may be explained by the sample size (only 50 hospital years for the correlation analysis with unnecessary care) and large variations in both unnecessary care and rates of cost recovery.

Table 6 shows the stepwise regression results for hospital revenue. The fitted model was statistically significant at the *p *< 0.001 level and could explain 86% of the total variation in hospital revenue (R^2 ^= 0.8636). Bonus type was selected into the model and was statistically significant (*p *< 0.01), explaining about 6% of the total variation in hospital service revenue. On average, as the parameter estimate shows, a switch to a bonus system with an expected stronger economic incentive increased hospital revenue by about two million yuan. The DEA efficiency score was not statistically significant and the unnecessary care indicator was not even selected into the model at the level of *p *= 0.15, implying that the relationship between revenue and productivity and the relationship between revenue and unnecessary care (as shown in the correlation analysis) are in fact the effect of the bonus type. In other words, bonus type may have affected hospital revenue through increases in hospital productivity and unnecessary care.

**Table 6:** Factors explaining the variation in hospital revenue (n = 50)

**Variable**	**Parameter estimate**	**F**	**P**	Partial R**2	Model R**2
INTERCEP	-99 008 144	63.24	0.0001		
Year	1 080 386	56.76	0.0001	0.7084	0.7084
Weishan	-4 029 314	13.42	0.0007	0.0708	0.7792
Bonus type	1 887 830	17.38	0.0001	0.0577	0.8369
Qixia	3 540 575	7.68	0.0082	0.0168	0.8357
DEA score	58 905	3.21	0.0800	0.0100	0.8636
Unnecessary care (not selected at P = 0.15 level)					

Model: F = 55.73 P = 0.0001 R^2 ^= 0.8636

As the partial R^2 ^shows, the year, which was selected with *p *= 0.0001, explained 70% of the total variation in hospital revenue. This is not surprising, given the change in revenue over time described earlier. The two hospital dummy variables jointly explained 9% of the revenue variation. These results imply that a dominant proportion of the revenue variation among years and hospitals can be explained by factors related to time and individual hospital characteristics. Although bonus type mattered, it could explain only a small portion of the variation.

Table 7 shows the stepwise regression results for rate of recovery of recurrent cost. The model was statistically significant (p < 0.001) with an R^2 ^of 0.6084. As with the regression on hospital revenue, the DEA efficiency score and unnecessary care were not selected into the model at the *p *= 0.15 level, and the bonus type was selected with a *p *value of 0.0129. The bonus type explained more than 3% of the variation in the rate of cost recovery, and on average a bonus switch to one with an expected stronger economic incentive contributed a two percentage-point increase to the rate of cost recovery. In contrast to the regression on hospital revenue, the year variable was no longer statistically significant and a dominant proportion (55%) of the variation in the rate of cost recovery was explained by hospital characteristics.

**Table 7:** Factors explaining the variation in hospital cost recovery (n = 50)

**Variable**	**Parameter estimate**	**F**	**P**	Partial R**2	Model R**2
INTERCEP	50.4365	1.58	0.2147		
Changyi	-24.1669	28.67	0.0001	0.1880	0.1880
Zhaoyuan	-19.2626	18.93	0.0001	0.1370	0.3250
Qixia	-23.7887	21.40	0.0001	0.1486	0.4736
Liangshan	-9.9650	5.87	0.0196	0.0818	0.5554
Bonus type	2.1861	6.38	0.0129	0.0306	0.5860
Year	0.6534	2.34	0.1336	0.0224	0.6084
DEA score (not selected at p = 0.15 level)					
Unnecessary care (not selected at p = 0.15 level)					

Model: F = 12.04 p = 0.0001 R^2 ^= 0.6084

Table 8 shows the stepwise regression results for unnecessary care. The model was statistically significant (*p *< 0.01) and the R^2 ^of the model was 0.3371. Bonus type was selected as a significant factor (*p *= 0.05) with a partial R^2 ^of 0.058, meaning that the difference in bonus system explained about 6% of the variation in unnecessary care. As the parameter shows, with a change in bonus type from non-bonus to flat or from flat bonus to revenue-related bonus, the unnecessary care indicator increased by about one percentage point – not as much as had been expected. After controlling for bonus type and hospital characteristics, the year and DEA efficiency score were not selected as significant factors explaining the difference in unnecessary care. The difference in characteristics of individual hospitals explained 27% of the total difference in unnecessary care and more than 60% of the variance was unexplained by this model.

**Table 8:** Factors explaining the variation in unnecessary care (n = 50)

**_Variable**	**Parameter estimate**	**F**	**P**	Partial R**2	Model R**2
INTERCEP	79.7257	2298.21	0.0001		
Liangshan	-5.3433	13.05	0.0007	0.1629	0.1629
Zhaoyuan	-3.6315	5.52	0.0232	0.1161	0.2790
Bonus type	1.0492	4.04	0.0582	0.0582	0.3372
Year (not selected at p = 0.15 level)					
DEA score (not selected at p = 0.15 level)					

Model: F = 7.80 P = 0.0003 R^2 ^= 0.3371

Table 9 shows the stepwise regression results for hospital productivity. The fitted model was statistically significant (*p *< 0.001) with an R^2^of 0.35. Unnecessary care was selected as a significant factor affecting hospital productivity, and the difference in unnecessary care explained 8.6% of the total variation in hospital productivity. A decrease in unnecessary care was associated with an increase in hospital productivity. This finding, and the fact that the DEA efficiency score was not selected as a significant factor explaining the difference in unnecessary care, imply that the relationship between unnecessary care and hospital productivity is such that the increase in unnecessary care reduced hospital productivity, rather than that the reduction in hospital productivity forced hospitals to provide more unnecessary care. Bonus type in this model was not found to be a significant factor, suggesting that the historical reduction in hospital productivity was not caused by switches in bonus system and that bonus switches failed to improve hospital productivity. Although the descriptive data showed that hospital productivity fell over time, year was not statistically significant in this model.

**Table 9:** Factors explaining the variation in hospital productivity (n = 50)

**Variable**	**Parameter estimate**	**F**	**P**	Partial R**2	Model R**2
INTERCEP	-55.8719	1.08	0.3038		
Year	-0.7421	2.89	0.0959	0.1075	0.1075
Unnecessary care	0.7010	4.10	0.0489	0.0859	0.1934
Zhaoyuan	14.6042	11.64	0.0014	0.0930	0.2864
Liangshan	6.2018	2.18	0.1468	0.0337	0.3201
Changyi	15.4109	12.83	0.0008	0.0321	0.3522
Bonus type (not selected at p = 0.15 level)					

Model: F = 12.05, P = 0.0001, R^2 ^= 0.3522

## Discussion

Given the absence of computerized information systems, this study was constrained by what data could feasibly be collected by hand. In particular this limited the study of unnecessary care, which was extremely time-consuming. If inpatient records had covered more years and more hospital years had been employed in the regression analyses, the factors that were not significant might have been statistically significant, and the goodness of fit of the models might have improved. Moreover, only two tracer conditions were studied, and only inpatient care was assessed. These conditions allow for only a limited degree of overprovision, especially in terms of lab tests and drugs, and so are likely to have underestimated the amount of unnecessary care.

Productivity assessment, for both unidimensional ratio analysis and DEA, had two major shortcomings: changes in the quality of care (other than the component of unnecessary care) were ignored, and changes in case mix were not adjusted for. Over time it is likely that the quality of care improved, and that case mix changed; thus this study is likely to have exaggerated the degree to which productivity declined over time. However, these points do not necessarily affect the finding that there was a lack of a statistically significant relationship between bonus type and hospital productivity. It is possible that hospital expansion and increased numbers of staff per hospital may have obscured any increase in quantity stimulated by the bonus payment.

The effect of bonus payment is likely to depend on both the type and the amount of bonus. However, data on the amount of bonus was a sensitive question, and panel hospitals either refused to provide data or provided data that were not considered reliable. In a related hospital census survey [18], it was found that there was no statistically significant difference in the average amount of bonus per doctor across the types of bonus. However, the variation of bonus payment among hospital doctors increased with the progression of bonus types. These findings suggest that exclusion of bonus amount in the analysis may not have introduced much error, because the average amount did not vary much, and it was the way in which the bonus was distributed that mattered.

In the analyses of individual hospitals and of trends, it was found that for some hospitals, the changes in indicators happened in the year of switch and for others the following year. There are two possible reasons. First, when hospitals responded to the bonus switch would depend on whether the bonus switch happened early or late in the year. Second, the speed of effects would also depend on how hospitals responded. Some may have responded quickly by admitting more patients, providing more existing services and prescribing more and costlier drugs. Others may have had to wait for the purchase of equipment (e.g. CT scanner) or the training of personnel (e.g. for open-heart surgery).

It was clear from the analysis that the increase in hospital cost recovery was not a result of improvement in hospital productivity, since between 1978 and 1997, cost recovery increased from 71% to 96% and the DEA efficiency score decreased from 97% to 73%. Apart from the increase in the proportion of unnecessary expenditure and the bonus system changes, there are at least four additional factors that may have affected the increase in hospital cost recovery.

First, over the previous 20 years the Chinese government had been raising medical care prices. Although these on average were set at about 50% of total cost [19], prices were higher relative to their cost than 10 years previously.

Second, due to the liberalization of the pharmaceutical market, the prices of drugs had increased considerably, doubling from 1980 to 1990 [8]. This benefited hospitals, because they were allowed to sell drugs at a 20% mark-up.

Third, the development of new technologies encouraged the introduction of new treatments and drugs that usually had much higher regulated prices than traditional treatments and drugs. For example, before 1980, imported drugs accounted for less than 1% of the Chinese drug market, while by 1993 the sale of imported drugs made up 30%–55% of the market in major cities such as Beijing and Shanghai [20]. Before 1980 there was no CT or MRI in China, while by 1995, CT scanners had became very popular in county hospitals and MRI could be found in any city at and above the prefecture level. Because the prices of high technology services could be set above cost, and the prices for the new imported drugs were 5 to 10 times the prices of the traditional and domestic drugs, hospitals obtained much more profit from using these services and drugs.

Finally, government budget reform increased the financial accountability of public hospitals. Reducing waste and saving costs became a major management concern, helping to improve hospital cost recovery. However, this study suggests that hospital characteristics explained around 55% of the variation in hospital cost recovery, implying that hospital management capacity and ability to control cost varied a great deal.

Between 1978 and 1997, the visits/admission ratio and admissions/operation ratio went down. The analysis showed that a bonus switch from one with a weaker economic incentive to one with a stronger incentive was one of the factors explaining the decreases in the visits/admission ratio and in the admissions/operation ratio, suggesting there must be other factors affecting these ratios. The number of visits to county hospitals was increasing until around the middle of the 1980s, and then started falling. Beginning at the end of the 1970s, rural economic reform brought about a rapid increase in peasants' income, which could have encouraged an increased demand for health care [21]. This may have been felt particularly at higher-level health institutions, such as county hospitals, because of the collapse of the rural Cooperative Medical System and the decrease in the number of rural doctors after the rural economic reform [22]. However, the increase in the number of rural doctors after the mid-1980s, when rural private practice was permitted, may have pulled patients back from county hospitals. In addition, the increase at that time in the number of county level health institutions, such as stations of maternal and child health and hospitals of Chinese traditional medicine, and the increase in medical prices, would have decreased the demand for county hospital care.

It is difficult to explain fully the steady increase in the number of admissions and the number of operations. Since inpatients can be admitted only through the outpatient department and only inpatients can be operated on, it is obvious that the hospitals admitted more and more from among the outpatients and performed more and more operations for the inpatients. There are several possible reasons for this. First, the case mix may have changed so that more patients needed to be admitted and operated on. Second, the development of technology and changes in medical criteria for admissions and operations may have led to more patients' being admitted and operated on. Third, the increase in the numbers of beds and doctors may have permitted needed admissions and operations that had not been possible before due to lack of inputs. Finally, related to the major hypothesis of this research, the changes in doctor payment system may have motivated staff to provide more unnecessary admissions and surgical operations. The study has shown that a bonus switch appeared to bring about an increase in the visits/admission ratio, but case notes did not permit a judgement as to whether an admission was necessary or unnecessary.

Although the DEA efficiency score decreased when panel hospitals switched from non-bonus to flat bonus, this does not mean that the bonus switch helped to decrease hospital productivity. This is, first, because the DEA efficiency score was generally decreasing over time; and second, because in two of the three hospitals (Liangshan and Changyi), the bonus switch appears to have helped to slow down the rate of decrease in the DEA efficiency score.

### Conclusions and policy implications

Based on these analyses, we can draw several conclusions. First, there was a steady increase in hospital revenue, and bonus type was a significant factor explaining its variation across hospitals and years.

Second, a considerable proportion of unnecessary expenditure out of total expenditure was identified, and there was a relationship between the bonus system and unnecessary care. Analyses showed that the bonus system was positively correlated with the unnecessary care indicator, implying that the higher the expected incentive of the bonus system, the higher the proportion of unnecessary expenditure.

Third, although hospital productivity decreased over time, a bonus switch from flat bonus to revenue-related bonus appeared to increase hospital productivity in the year of the switch. A bonus switch from non-bonus to flat bonus was not similarly able to reverse the trend of hospital productivity, but it seemed that the rate of decrease in hospital productivity was slowed down by a bonus switch.

Fourth, hospital cost recovery increased over time. The study suggests that the bonus switch brought about an increase in cost recovery and that the bonus system was positively correlated with hospital cost recovery.

Last and in general, the research suggests that the bonus change over time contributed significantly to the increase in hospital service revenue and hospital cost recovery. The increase in unnecessary care and increase in the number of admissions out of the existing number of outpatients, with the bonus system switching from one with a weaker incentive to one with a stronger incentive, suggests that the improvement in hospital cost recovery was achieved at least in part through the provision of more unnecessary care and drugs and through admitting more patients.

There are two policy implications from this study. First, there is little evidence that the performance-related pay system as designed by Chinese public hospitals is socially desirable. It could improve hospital financial sustainability, but did not necessarily lead to improvements in efficiency from a social perspective. The key barrier to achieving the social objectives of performance-related pay was the inappropriate link (whether direct or indirect) between bonus payment and hospital revenue. Hospital bonus distribution should be based on doctor performance measured by indicators that are in line with the desired overall performance of the health care system.

Second, reforms in various countries are characterized by increased exposure of public hospitals to financial risk, in order to increase financial accountability, efficiency and productivity. However there is a risk of encouraging revenue maximization and rent-seeking. Chinese experiences show that when increasing public hospital autonomy, hospitals should be monitored closely by the government, and regulations applied to limit opportunistic behaviour. Otherwise, the containment of government financing to public facilities may result in an increase in the provision of unnecessary care, an increase in health costs to society and a waste in social resources.

## Competing interests

The author(s) declare that they have no competing interests.

## Authors' contributions

XL designed the study, carried out the fieldwork, undertook the data analysis and wrote the initial draft. AM guided and supervised the design and data analysis and participated in writing and finalizing the paper. Both authors read and approved the final manuscript.

## References

[B1] Sharma S, Hotchkiss DR (2001). Developing financial autonomy in public hospitals in India: Rajasthan's model.. Health Policy.

[B2] Preker AS, Harding A (2003). Innovations in service delivery.

[B3] Health M (1997). The resolution of strengthening health work..

[B4] Chang SC (1986). Does the hospital responsibility system increase the social benefit of hospital services?. Chinese Health Economics.

[B5] Jiang GH (1990). The reasons for the reduction in the effect of bonus system and related countermeasures.. Chinese Health Economics.

[B6] Yi QY (1990). The mistakes of hospitals' motivation mechanism.. Chinese Health Economics.

[B7] Wang GZ, Lin SY (1992). The health economic problems of bonus system.. Chinese Health Economics.

[B8] Li Q (1997). The prospect of the Chinese pharmaceutical market in 1996.. Chinese Pharmaceutical Information.

[B9] Bian ZP (1994). Reforming hospital management and the income distribution of medical staff.. Chinese Health Economics.

[B10] Zhang MN, Lin YY (1994). The fairness and efficiency of bonus distribution.. Chinese Health Economics.

[B11] Han T, Qiao TY, Cui P (1994). An effective way of designing the bonus system.. Chinese Health Economics.

[B12] Jiang DJ (1995). Reforming the distribution mechanism and increase the activeness of the hospitals.. Chinese Health Economics.

[B13] Liu X (1999). Impact of payment reforms to hospital-based doctors on the efficiency of hospital services: a study of county general hospitals in China.  PhD thesis..

[B14] Sherman HD (1984). Hospital efficiency measurement and evaluation.. Medical Care.

[B15] Valdmanis V (1990). Ownership and technical efficiency of hospitals.. Medical Care.

[B16] Thanassoulis E, Emrouznejad A (1998). Warwick Windows DEA User's Guide.

[B17] Liu X, Mills A (1999). Evaluating payment mechanisms: how can we measure unnecessary care.. Health Policy and Planning.

[B18] Liu X, Mills A (2003). The influence of bonus payments to doctors on hospital revenue: results of a quasi-experimental study. Applied Health Economics and Health Policy.

[B19] Health HDP (1993). The current status of medical price management and its reform.. Chinese Journal of Health Economics.

[B20] Zhang WK (1992). The further improvement of drugs administration and inspection.. Chinese Pharmacy.

[B21] Hsiao WC (1987). Transformation of health care in China.. New England Journal of Medicine.

[B22] Liu X, Cao H (1992). The Chinese cooperative medicine: its historical transformation and the future trend of development. Journal of Public Health Policy.

